# Educational Disparities in COVID-19 Prevention in China: The Role of Contextual Danger, Perceived Risk, and Interventional Context

**DOI:** 10.3390/ijerph18073383

**Published:** 2021-03-24

**Authors:** Miao Li, Weidong Wang

**Affiliations:** 1 Department of Sociology, Anthropology, and Criminal Justice, Clemson University, 135A Brackett Hall, Clemson, SC 29634, USA; miaol@clemson.edu; 2 Department of Sociology, Renmin University of China, 59 Zhongguancun St., Beijing 100872, China

**Keywords:** COVID-19, disparities, distancing, education, fundamental cause, mask-wearing, hand hygiene

## Abstract

Despite the social disparities in COVID-19 infection, little is known about factors influencing social disparities in preventive behaviors during the pandemic. This study examined how educational disparities in mask-wearing, handwashing, and limiting public outings might be contingent upon three factors: contextual cue of danger, perceived risk of local outbreak, and interventional context with different levels of intensity (i.e, Wuhan vs. other areas). Data were obtained from a telephone survey of 3327 adults, who were recruited through a random-digit-dial method to be representative of all cell phone users in China. Interviews were conducted from 28 April to 26 May 2020. Stratified multiple regression models showed that educational disparities in all three behaviors were only consistently observed among people exposed to context cues of danger, with an enhanced sense of risk of a local outbreak, or in areas other than Wuhan. College education seems to make a difference in handwashing regardless of contextual cues of danger or perception of risk. The findings suggested that, in the process of an epidemic, emerging threats in one’s immediate environment or raised awareness of risks are important conditions triggering educational disparities in prevention. However, effective public health interventions could potentially reduce such disparities.

## 1. Introduction

Multiple studies have documented the social disparities in COVID-19 infection, testing, and death [1,2,3]. In the absence of vaccine or treatment medications, preventive measures such as mask-wearing, hand hygiene, and social distancing become the only effective options in curbing the spread of the disease [4,5]. As such, examining social disparities in these preventive behaviors will help us understand social disparities in COVID-19 infection. Moreover, as the pandemic continues to progress, it is urgent to understand what factors may promote or reduce such disparities.

Built upon the large body of literature documenting the strong and consistent associations between education and health behaviors [6,7,8,9], this study examined whether educational disparities in mask-wearing, handwashing after a public outing, and limited public outings are contingent upon the following social factors: contextual cues of danger in the immediate environment, perceived risk of local outbreak, and intensive public health intervention.

### Theory and Hypotheses

The fundamental cause theory (FCT) suggests that people with higher socioeconomic status (SES) are more likely to engage in healthful behaviors because of their access to flexible resources such as knowledge, money, power, prestige, and beneficial social connections [10]. Education is a unique dimension of SES that precedes and generates other flexible resources [11]. Compared to material resources or monetary goods, education is an intra-personal resource that has a more direct link with human agency and, therefore, is more relevant for behaviors [12,13]. For example, education has direct and profound impacts on one’s lifestyle, social network, mastery, reasoning ability, problem-solving ability, decision making, motivation, and values [14,15,16,17]. The human and material capital bestowed by education could readily be translated into health-protective behaviors [18,19,20].

The FCT identified conditions that may either promote or weaken social disparities in health behaviors. Specifically, conditions promoting relevance or enactment of individual resources will strengthen social disparities in health [21]. The emergence of risk information, such as contextual cues for danger and enhanced perception of risk, would promote the enactment/mobilization of personal resources in responding, thus providing an ideal condition for social disparities in preventive behaviors to emerge [16,21]. For example, the educational disparity in smoking began to manifest and grew stronger as the information on hazards of smoking was continually disseminated among the U.S. population over time, because people of higher education were more likely and quicker to respond to such information (e.g., less likely to start smoking and more likely to quit) [22,23,24]. Similarly, the educational disparity in cocaine use suddenly emerged in a brief period of the 1980s, when cocaine was redefined from being harmless and recreational to being dangerous, unhealthy, and crime-breeding [25,26]. The risk information on cocaine use was more readily taken advantage of by the better-educated people who were more able to change their behavior and stop using cocaine. 

In the context of the COVID-19 pandemic, the emergence of threat in the immediate environment (such as contraction or suspected contraction within one’s social network and/or neighborhood), or enhanced individual perception of imminent risk of a local outbreak (either with or without the presence of threatful cues in the immediate environment), are important factors triggering the mobilization of personal resources in health-related decision-making and accordingly, would facilitate the emergence of educational disparities in health behaviors. Given the contextual cue of danger or enhanced perception of risk, more educated people might have a better understanding of the situation and have more resources and/or competence in initiating and sustaining a behavioral change [27]. The more-educated might also be more motivated to practice prevention given that they tend to have less fatalistic beliefs and higher opportunity costs of failing health [28,29]. Indeed, other behavioral theories such as the theory of planned behaviors suggested that education represents a critical factor in shaping a person’s social norm exposure, behavioral intention, perceived behavioral control, and actual behavioral change [30]. As such, we hypothesize that (1) educational disparities in the above preventive behaviors are more likely to exist among people in an environment with cues for danger, and (2) educational disparities in these preventive behaviors are more likely to exist among people with an enhanced sense of risk.

On the other hand, conditions suppressing the enactment of individual resources will weaken social disparities in health [21]. For example, educational disparities are much smaller or nonexistent in health conditions that are less or not preventable, in which case people of higher education cannot employ flexible resources to gain any special advantages [31,32,33]. Other than the nature of the health conditions, the FCT argues that effective public health interventions that reduce the relevance of personal resources could also reduce the educational disparities in health behaviors [21]. Existing intervention studies have garnered some promising evidence in this respect. For instance, income enhancement and supplement programs could reduce the educational disparities in maternal smoking [34]. Multifaceted interventions integrating nutrition parties, debt assistance, improved access to walking and cycling routes, daily TV guided-aerobics programs, and antismoking campaigns could reduce the educational disparities in fat consumption and some physical activities [35]. 

In the COVID-19 outbreak, intensive interventions could greatly prompt and enable people of all social backgrounds to adopt effective preventive behaviors, thus reduce educational disparities. Examples of such interventions include face mask ordinance, massive donation and redistribution of relief supplies, *cordons sanitaire*, traffic restriction, organized and rapid scaling up of the e-commerce infrastructure to reduce public outings, and mass media and community campaigns promoting hand hygiene and social distancing rules/methods [36]. During the COVID-19 outbreak in China, the city of Wuhan, the ground zero and epicenter of the pandemic, has gone through more intensive public health interventions than other places, supported by an influx of resources from all over the country. Other than the 76-day city lockdown, the above-mentioned intervention strategies were more thoroughly and more rigorously (sometimes even forcefully) implemented in Wuhan than in other places, where the interventions are mainly focused on preventing importations [37]. Such a situation provided a unique opportunity to observe how educational disparities may vary across interventional contexts. Based on the FCT, we hypothesize that educational disparities in these preventive behaviors are less likely to be observed in Wuhan than in other places. 

## 2. Materials and Methods

### 2.1. Data

The Chinese Survey of COVID-19 Impact-Wave I (CSCI-I) is a national cell phone survey of 4653 adults living in 31 provinces, municipalities, or autonomous regions in mainland China. The CCIS-I also included 1385 oversampled individuals living in Wuhan, the epicenter of COVID-19, in order to obtain a large enough number of cases with which to conduct a meaningful statistical comparison. The purpose of the CCIS is to collect longitudinal survey data on the social, economic, behavioral, and psychological impacts of COVID-19 on the Chinese population. Data collection for this study was approved by the Institutional Review Board of Renmin University of China (Approval Number: RN20200401). Informed consent was obtained from all individual participants included in the study. The survey was conducted from 28 April to 26 May 2020 by researchers at the National Survey Research Center at the Renmin University of China. A team of trained staff conducted the phone interviews following the same protocol and interview schedule. 

Using the random-digit-dial (RDD) method, a sample of randomly generated cell phone numbers was selected to be representative of all cell phone users in China. According to the statistics from the Ministry of Industry and Information Technology of the People’s Republic of China, the mobile phone coverage rate is 113.9 cell phones per hundred people [38]. Of 15,032 individuals contacted, 4653 completed the phone interview (response rate = 31%). The sample was weighted to population benchmarks on several sociodemographic dimensions such as sex, age, and education. After listwise deletion of individuals with missing data on any variables involved in the analyses, the final analytic sample for this study included 3327 individuals (missing rate = 28%). Because of the high missing rate, sensitivity analyses were conducted based on 20 multiply imputed data sets, the results of which are presented in the Supplementary Materials. 

### 2.2. Measurements

Dependent Variables. The study examined three preventive behaviors: mask-wearing in public, handwashing after a public outing, and limited public outings. Study participants’ self-reported frequencies of these preventive behaviors were measured on a 5-point scale, ranging from “not at all” to “all the time”, with higher values representing higher frequency. The original survey questions asked were “how often are you currently practicing the following preventive measures: mask-wearing in public, handwashing after a public outing, and limited public outings”. Survey questions for the dependent variables, together with questions measuring other relevant variables, are summarized in Table A1 (Appendix A).

Independent Variable. Self-reported level of education was measured in three categories: “high school or lower”, “associate degree”, and “bachelor’s degree or above”. The original response categories for the survey question on education are “middle school or below”, “high school or vocational school”, “associate degree”, “bachelor’s degree”, and “postgraduate degree”. Given the small percentage of the population with graduate degrees (<1%), our coding followed the common practice of combining the “bachelor’s degree” and the “graduate degree” [39,40,41]. We kept the “associate degree” as a separate category as the associate degree education (i.e., zhuanke, is often offered in 2- or 3-year college courses) is an important component of the Chinese higher education system and, therefore, is represented as a separate category in major social surveys, such as the Chinese General Social Survey. Additionally, our preliminary analyses suggested that this category is significantly different from lower educational categories (i.e., “middle school or below” and “high school or vocational school”) in the focal preventive behaviors. Similarly, we combined the “middle school or below” and the “high school or vocational school” based on our preliminary analyses showing that these two categories do not significantly differ in the focal preventive behaviors.

Moderator. The study examined three factors that were hypothesized to influence the educational disparities in preventive behaviors mentioned above: contextual cue of danger, perceived risk of a local outbreak, and interventional context. The contextual cues of danger were measured based on three survey questions asking (1) whether the participants had any family members tested positive or (2) quarantined/hospitalized due to suspected coronavirus positivity, and (3) whether any COVID-19 cases were reported in the neighborhood/village that they are living in. A global dichotomous measure was constructed with 1 representing an endorsement of any of the above situations. Perceived risk of the outbreak was measured with a survey question asking respondents to rate their perceptions of risk for an imminent COVID-19 outbreak in the area they live in. Responses were dichotomized with 1 representing “medium or high risk” and 0 “zero or low risk”. The interventional context was operationalized as a dummy variable differentiating Wuhan from other places. 

Covariates. The study adjusted for the following variables: sex (male, female), age (in years), urbanicity (urban, rural), average income earned by each person within the household (unit: in logged ten thousands ¥), occupation (executives or professionals, unskilled or low-skilled labor, currently not in the labor force, other), self-rated social ranking, and individual coronavirus exposure. The self-rated social ranking was measured along a 5-point scale indicating “lower class”, “lower middle class”, “middle class”, “upper middle class”, and “upper class”, with a higher value indicating higher perceived social ranking compared to other people within the local community. Individual coronavirus exposure was measured as a dummy variable with 1 representing that the participant had been either tested positive or quarantined/hospitalized due to suspected coronavirus positivity. 

### 2.3. Statistical Analyses

Analyses were conducted with Stata, version 14 (StataCorp LP, College Station, TX, U.S.) in July‒August 2020. First, weighted sample characteristics were reported across interventional contexts and educational groups. Significance tests for educational disparities in sample characteristics were performed using χ^2^ tests for categorical variables and *t*-tests for continuous variables. Then, a series of multiple linear regressions respectively stratified by a contextual cue of danger, perceived risk of a local outbreak, and interventional context was conducted to predict the frequencies of the aforementioned preventive behaviors, adjusting for covariates listed above. Finally, to further reduce the sample heterogeneity in comparison, we re-estimated the above models using only the urban subsample in a series of sensitivity analyses. We also re-estimated the moderation effects of the contextual cues of danger and perceived risk using the subsample taken from outside of Wuhan. Significance levels in all analyses were set at 0.05 and all tests were two-sided.

## 3. Results

Table 1 presents the weighted sample characteristics stratified by interventional context and educational level. Across different interventional contexts (i.e., Wuhan vs. other areas), we found that people in Wuhan were (1) more likely to report coronavirus positive or suspected positive, (2) more likely to report COVID-19 cases or suspected cases in family/neighborhood, (3) had a higher perceived risk of a local outbreak, and (4) practiced more frequently all three types of preventive behaviors than people in other areas. In terms of demographics, the study sample in Wuhan was on average younger, more educated, had more prestigious occupations, had higher income, and self-rated social rank. The educational disparity in mask-wearing existed in both Wuhan and other areas, while the educational disparity in handwashing only existed in other areas. There is no educational disparity in limiting public outings either in Wuhan or other areas. 

Table 2 presented regression models for three preventive behaviors. For each outcome, the models were stratified by the context cue of danger, perceived risk of a local outbreak, and interventional context, the results of which were respectively summarized in the upper, middle, and lower panel of the table. In general, consistent with our hypotheses, the associations between education and all three preventive behaviors were more pronounced with the presence of contextual danger, enhanced perceived risk, and without intense public health interventions (i.e., areas other than Wuhan).

The upper panel in Table 2 showed that with the presence of the context cues of danger (i.e., family members tested positive or suspected positive, COVID-19 cases reported in the neighborhood/village), education was significantly positively associated with all three preventive behaviors: people with associate degrees or bachelor’s degrees or above practiced all three outcomes more often than people with high school or lower education. Without the presence of contextual cues of danger, however, education was not associated with mask-wearing or limited public outings and was associated with handwashing only at the bachelor’s degree or above level. 

The middle panel of Table 2 showed that for people with medium/high perceived risk for s local outbreak, education was significantly positively associated with all three preventive behaviors. For people with low/no perceived risk of an outbreak, however, education was not associated with mask-wearing or limited public outings and was weakly associated with handwashing only at the bachelor’s degree or above level. 

The lower panel of Table 2 indicated that education was not associated with any behavioral outcomes for people in Wuhan, which had gone through intensive public health interventions by the time of the survey. Outside of Wuhan, people with bachelor’s degrees or above practiced all three preventive behaviors more often than those with high school or lower education. Associate degrees, however, did not make any difference in preventive behaviors in either Wuhan or other places.

As most study participants in Wuhan lived in the urban setting, to increase comparability between the Wuhan sample and the sample from other areas, we conducted sensitivity analyses in which all models were re-estimated excluding all individuals who reported to currently living in rural settings (i.e., using only the urban subsample). Results from such analyses yielded quite similar patterns (Table 3). 

By the time of the survey, the epidemic was largely under control in China. However, sporadic cases of infection were still being reported throughout the country, and fear of a local outbreak was still felt by many people. To explore how contextual cues of danger and perceived risk of a local outbreak may influence educational disparities in preventive behaviors in a context that has not gone through intensive interventions as those enforced in Wuhan, we conducted exploratory analyses using only the subsample from outside of Wuhan. Results (Table 4) suggested similar patterns as were reported in Table 2. Notably, in these areas, the educational disparities appeared to be even more pronounced given the presence of context cues of danger or medium/high perceived risk of a local outbreak, as was indicated by the generally larger coefficient estimates for associate degrees and bachelor’s degrees or above than those in the main analyses (i.e., Table 2). 

## 4. Discussion

In line with our hypotheses, the study found that education was positively associated with all three preventive behaviors in the presence of contextual cues of danger, enhanced perceived risk, and in areas other than Wuhan. In the absence of contextual cues of danger or for people with low/no perceived risk, education was not associated with mask-wearing or limited public outings and was weakly associated with handwashing only at the bachelor’s degree or above level. Education was not associated with any preventive behaviors in Wuhan.

These findings could be understood in light of the fundamental cause theory (FCT). According to the FCT, educational disparities in preventive behaviors are likely to manifest in circumstances under which personal resources become more relevant. The emergence of risk information, be it contextual cues of danger or perceived risk of a local outbreak, is often the first motivator for adopting prevention measures during an infectious disease outbreak. However, insofar as such risk information triggers the mobilization of personal resources, which is unequally distributed in the population, educational disparities in health behaviors are likely to emerge. In contrast, no educational disparities were observed in mask-wearing or limited public outings without contextual cues of danger or enhanced perception of risk. 

The null associations between education and all three preventive behaviors in Wuhan suggested that intensive multifaceted public health interventions could override the inequality-generating effect from the individualistic reactions. By the time of the survey, Wuhan has gone through much more intensive public health interventions than anywhere in the country, such as, systematically enforced face mask ordinance, massive distribution of masks, compulsory stay-at-home policies, *cordon sanitaire*, organized scaling-up of e-commerce, and more importantly, the city lock-down. All of these measures, together with their huge psychological impacts, could generate a leveling effect on the educational disparities by equalizing access to knowledge, risk information, and material/motivational capital.

A unique finding that is inconsistent with our hypotheses is about the educational disparity in handwashing: people with bachelor’s degree or above practice handwashing more often than those with high school or lower, regardless of the contextual threat and perceived risk of a local outbreak. A speculated explanation for such a pattern is that, compared to mask-wearing and limited public outings, handwashing after a public outing is a common hygiene practice that is not unique to dealing with COVID-19 and, accordingly, the educational disparity in such preventive behavior may not be very sensitive to COVID-9 related cues or risks.

Given the recentness of the pandemic and the tremendous difficulties it poses for data collection, we have not located any published studies that explicitly examined social factors that may modify the education-behavior associations during the COVID-19 pandemic. This makes our contribution timely and unique. However, our findings of the educational disparities in preventive behaviors in areas other than Wuhan are consistent with studies from other countries, such as Germany and Saudi Arabia, where the educational disparities in prevention were also observed [42,43].

The findings of this study provided some important insights that could inform public responses to the COVID-19 pandemic. First, our findings imply that educational disparities in preventive behaviors are more likely to manifest at the early stage of the community-level outbreak (or when the disease is entering into a new area), as the emerging threat in the environment and/or the enhanced sense of risk of an outbreak within the population immediately calls for individual responses (based on personal resources) when public interventions may take time to be implemented or to function. Our findings also suggest that interventional strategies that only focus on promoting risk awareness are likely to promote educational disparities in prevention. An enhanced sense of risk will not automatically translate into preventive behaviors, and education plays an important role in either facilitating or blocking such translation. Unfortunately, however, it is not uncommon that public health interventions in many parts of the world stopped at awareness promotion. Finally, our findings suggest that interventions addressing underlying social and environmental conditions could reduce educational disparities in prevention. With that being said, we have noticed that the intensive intervention measures in Wuhan involved violations of individual rights (i.e., forced quarantine, contract tracing intruding personal privacy, etc.). We are certainly not recommending extreme policies as such. But difficulties in data collection created by the COVID-19 pandemic limited our capacity in evaluating interventions that successfully reduced inequality without significant costs on individual rights. 

A few limitations should be considered in interpreting the findings. First, CSCI-I had a low response rate, which is very common for telephone surveys. Such design was chosen because of the extreme difficulties in fielding face-to-face interviews given the current health concerns with COVID-19. However, methodological studies suggested that nonresponse bias may not necessarily be higher for surveys with low response rates and short field periods than for surveys with high response rates and long field periods [44]. Second, even though the mobile phone coverage rate is very high in China, it is possible that some people from certain vulnerable groups (e.g., the elderly or uneducated) may not have access to mobile phones and thus were excluded from the sampling frame. Given that education was positively associated with preventive behaviors, the exclusion of the low-educated population could potentially lead to model underestimation, at least under conditions promoting the relevance of personal resources (i.e., presence of contextual danger cues, enhanced perceived risk, and without intensive intervention). Third, we relied on self-reported measurements for our focal outcomes, the validity of which is not very strong. Finally, this study only focused on preventive behaviors, not the outcome. Even with a large number of infections, confirmed COVID-19 cases still are rare events that could hardly be captured in sampling. The very few COVID-19 confirmed cases within the sample therefore do not support any meaningful analyses. 

## 5. Conclusions

This is the first study examining circumstances or conditions that may either promote or reduce educational disparities in three important COVID-19 prevention behaviors: mask-wearing in public, handwashing after public outings, and limited public outings. It found that education was positively associated with all three preventive behaviors with the presence of contextual cues of danger, enhanced perceived risk, and without intense public health interventions (i.e., areas other than Wuhan). With the absence of contextual cues of danger or with low/no perceived risk, however, education was not associated with mask-wearing or limited public outings and was associated with handwashing only at the bachelor’s degree or above level. Education was not associated with any preventive behaviors in Wuhan. 

Despite the limitations, this study provided important evidence regarding educational disparities in COVID-19 preventive behaviors, as well as circumstances in which such disparities may emerge or be mitigated. Its findings would inform equitable interventions aimed at reducing social disparities in COVID-19 outcomes. Meanwhile, the study also calls for additional studies in other parts of the world with a goal for evaluating social conditions/policies that may either promote or reduce social disparities in COVID-19. 

## Figures and Tables

**Table 1:** Weighted mean ± sd or % of the study sample, The Chinese Survey of COVID-19 Impacts 2020 (*N* = 3327).

		Wuhan (*N* = 1038)					Other Areas (*N* = 2289)				*p*-Value ^b^
	Total	Level of Education			*p*-Value ^a^	Total	Level of Education			*p*-Value ^a^	
		High School or Lower (79.9)	Associate Degree (7.2)	Bachelor’s Degree or Above (12.9)			High School or Lower (85.96)	Associate Degree (5.0)	Bachelor’s Degree or Above (9.1)		
**Outcomes**											
Mask Wearing	3.8 ± 0.4	3.8 ± 0.4	3.9 ± 0.3	3.9 ± 0.4	0.019	3.6 ± 0.7	3.6 ± 0.7	3.6 ± 0.6	3.6 ± 0.6	0.000	0.000
Handwashing	3.7 ± 0.5	3.7 ± 0.5	3.8 ± 0.5	3.8 ± 0.5	0.058	3.6 ± 0.7	3.5 ± 0.7	3.6 ± 0.7	3.6 ± 0.6	0.002	0.000
Limited Public Outing	3.5 ± 0.7	3.5 ± 0.7	3.5 ± 0.7	3.4 ± 0.8	0.818	3.4 ± 0.8	3.3 ± 0.8	3.2 ± 0.8	3.3 ± 0.8	0.083	0.000
**Covariates**											
Male	42.9	39.9	55.4	54.5	0.000	51.5	50.9	58.4	53.3	0.181	0.000
Age	48.1 ± 2.6	50.9 ± 16.2	39.7 ± 14.0	35.7 ± 12.3	0.000	43.9 ± 15.8	45.7 ± 15.7	35.4 ± 12.7	31.4 ± 11.5	0.000	0.000
Urban	87	85.2	93.8	94.2	0.000	53.7	48.6	82.9	85.3	0.000	0.000
Occupation											
*Executives/professionals*	10.5	4.3	20.8	42.7	0.000	7.8	3.2	24.7	41.9	0.000	0.003
*Unskilled or low-skilled labor*	46.4	48.5	47.6	32.4		60.0	64.2	48.6	25.9		
*Not in the labor force*	36.7	40.9	23.0	18.3		26.2	26.8	17.9	25.5		
*Other*	6.5	6.3	8.6	6.6		6.0	5.8	8.8	6.7		
Average annual individual income within household (in ten thousand)	3.3 ± 4.2	2.5 ± 3.0	4.2 ± 3.8	7.5 ± 7.2	0.000	2.8 ± 4.4	2.3 ± 3.6	4.6 ± 6.9	6. ± 7.2	0.000	0.001
Subjective Social Ranking	2.4 ± 0.8	2.3 ± 0.8	2.7 ± 0.6	3.0 ± 0.6	0.000	2.4 ± 0.85	2.3 ± 0.9	2.7 ± 0.7	2.8 ± 0.7	0.000	0.029
Individual Coronavirus Positive or Suspected Positive	2.8	2.6	2.1	4.3	0.369	0.8	0.6	1.4	1.8	0.046	0.001
Context Cue of Danger: Yes	56.4	51.7	75.7	75.2	0	6.0	4.9	11.9	13.2	0	0.000
Perceived Local Risk of COVID-19 Outbreak: Medium/High	18.5	17.3	18.7	25.4	0.086	9.0	9.3	8.1	6.7	0.295	0.000

^a^*p*-values were based on chi-squared tests for categorical variables or *t*-tests for continuous variables across educational levels; ^b^*p*-values were based on chi-squared tests for categorical variables or *t*-tests for continuous variables between Wuhan and Other Areas.

**Table 2:** Educational Disparities in COVID-19 Preventive Behaviors Stratified by Context Cues of Danger, The Chinese Survey of COVID-19 Impacts 2020 (*N* = 3327).

Context Cue of Danger	Mask Wearing ^a^		Handwashing ^a^		Limited Public Outing ^a^	
	Present (*N* = 2171)	Absent (*N* = 1156)	Present (*N* = 2171)	Absent (*N* = 1156)	Present (*N* = 2171)	Absent (*N* = 1156)
Intercept	3.54 ***	3.63 ***	3.36 ***	3.13 ***	3.27 ***	3.08 ***
	(3.41, 3.67)	(3.48, 3.77)	(3.22, 3.50)	(2.94, 3.31)	(3.09, 3.44)	(2.81, 3.34)
Education ^b^						
*≤High School*						
*Associate Degree*	0	0.10 **	0.04	0.10 *	−0.04	0.18 **
	(−0.08, 0.07)	(0.03, 0.17)	(−0.04, 0.12)	(0.01, 0.19)	(−0.14, 0.06)	(0.06, 0.31)
*≥Bachelor’s Degree*	0.02	0.12 ***	0.10 *	0.12 **	0.05	0.15 *
	(−0.06, 0.09)	(0.05, 0.19)	(0.02, 0.18)	(0.04, 0.21)	(−0.05, 0.15)	(0.02, 0.27)
**Perceived Risk of Local Outbreak**	Zero/Low (*N* = 2870)	Medium/High (*N* = 457)	Zero/Low (*N* = 2870)	Medium/High (*N* = 457)	Zero/Low (*N* = 2870)	Medium/High (*N* = 457)
Intercept	3.55 ***	3.43 ***	3.31 ***	3.14 ***	3.18 ***	3.16 ***
	(3.44, 3.66)	(3.20, 3.65)	(3.19, 3.42)	(2.84, 3.44)	(3.03, 3.33)	(2.74, 3.59)
Education ^c^						
*≤High School*						
*Associate Degree*	0.01	0.17**	0.04	0.24 **	−0.01	0.42 ***
	(−0.05, 0.07)	(0.05, 0.30)	(−0.03, 0.10)	(0.07, 0.41)	(−0.09, 0.07)	(0.18, 0.67)
*≥Bachelor’s Degree*	0.03	0.20 **	0.09 **	0.22 **	0.04	0.36 **
	(−0.03, 0.09)	(0.08, 0.33)	(0.03, 0.16)	(0.06, 0.38)	(−0.04, 0.13)	(0.12, 0.59)
**Interventional Context**	Wuhan (*N* = 1038)	Other Areas (*N* = 2289)	Wuhan (*N* = 1038)	Other Areas (*N* = 2289)	Wuhan (*N* = 1038)	Other Areas (*N* = 2289)
Intercept	3.71 ***	3.51 ***	3.24 ***	3.30 ***	3.12 ***	3.22 ***
	(3.57, 3.85)	(3.38, 3.64)	(3.04, 3.43)	(3.16, 3.44)	(2.84, 3.39)	(3.05, 3.39)
Education ^d^						
*≤High School*						
*Associate Degree*	0.02	0.04	0.08	0.05	0.04	0.04
	(−0.05, 0.08)	(−0.03, 0.11)	(−0.01, 0.18)	(−0.03, 0.13)	(−0.09, 0.17)	(−0.05, 0.14)
*≥Bachelor’s Degree*	−0.02	0.08 *	0.04	0.14 ***	−0.01	0.12 *
	(−0.09, 0.04)	(0.01, 0.16)	(−0.06, 0.13)	(0.07, 0.22)	(−0.14, 0.12)	(0.03, 0.22)

^a^ All models adjusted for sex, age, urbanicity, income, occupation, subjective social ranking, and individual coronavirus exposure. ^b^ Models additionally adjusted for the perceived risk of outbreak and interventional context. ^c^ Models additionally adjusted for context cue of danger and interventional context. ^d^ Models additionally adjusted for context cue of danger and perceived risk of an outbreak. 95% confidence intervals in brackets. * *p* < 0.05, ** *p* < 0.01, *** *p* < 0.001.

**Table 3:** Educational Disparities in COVID-19 Preventive Behaviors Stratified by Context Cues of Danger, The Subsample of Urban Residents, The Chinese Survey of COVID-19 Impacts 2020 (*N* = 2565).

Context Cue of Danger	Mask Wearing ^a^		Handwashing ^a^		Limited Public Outing ^a^	
	Present (*N* = 1514)	Absent (*N* = 1051)	Present (*N* = 1514)	Absent (*N* = 1051)	Present (*N* = 1514)	Absent (*N* = 1051)
Intercept	3.75 ***	3.69 ***	3.38 ***	3.28 ***	3.04 ***	2.86 ***
	(3.61, 3.90)	(3.55, 3.83)	(3.21, 3.55)	(3.11, 3.46)	(2.82, 3.25)	(2.59, 3.12)
Education ^b^						
*≤High School*						
*Associate Degree*	−0.04	0.09 *	0.02	0.12 **	−0.05	0.18 **
	(−0.12, 0.03)	(0.02, 0.17)	(−0.07, 0.11)	(0.03, 0.21)	(−0.17, 0.06)	(0.04, 0.32)
*≥Bachelor’s Degree*	0.01	0.10 **	0.11 *	0.14 **	0.06	0.14 *
	(−0.07, 0.08)	(0.03, 0.17)	(0.02, 0.20)	(0.06, 0.23)	(−0.06, 0.17)	(0.01, 0.28)
**Perceived Risk of Local Outbreak**	Zero/Low (*N* = 2183)	Medium/High (*N* = 382)	Zero/Low (*N* = 2183)	Medium/High (*N* = 382)	Zero/Low (*N* = 2183)	Medium/High (*N* = 382)
Intercept	3.72 ***	3.62 ***	3.32 ***	3.30 ***	2.93 ***	3.09 ***
	(3.61, 3.83)	(3.39, 3.85)	(3.19, 3.45)	(3.00, 3.61)	(2.75, 3.10)	(2.61, 3.56)
Education ^c^						
*≤High School*						
*Associate Degree*	−0.01	0.15 *	0.04	0.23 **	−0.02	0.43 **
	(−0.07, 0.05)	(0.02, 0.28)	(−0.03, 0.11)	(0.06, 0.41)	(−0.11, 0.07)	(0.16, 0.70)
*≥Bachelor’s Degree*	0.01	0.22 ***	0.10 **	0.26 **	0.04	0.37 **
	(−0.04, 0.07)	(0.10, 0.34)	(0.03, 0.17)	(0.09, 0.43)	(−0.05, 0.13)	(0.11, 0.63)
**Interventional Context**	Wuhan (*N* = 935)	Other Areas (*N* = 1630)	Wuhan (*N* = 935)	Other Areas (*N* = 1630)	Wuhan (*N* = 935)	Other Areas (*N* = 1630)
Intercept	3.91 ***	3.67 ***	3.39 ***	3.30 ***	3.07 ***	2.96 ***
	(3.78, 4.04)	(3.53, 3.82)	(3.20, 3.57)	(3.14, 3.46)	(2.79, 3.34)	(2.74, 3.17)
Education ^d^						
*≤High School*						
*Associate Degree*	0	0.02	0.07	0.06	0.05	0.04
	(−0.06, 0.07)	(−0.06, 0.09)	(−0.02, 0.16)	(−0.03, 0.14)	(−0.09, 0.19)	(−0.07, 0.16)
*≥Bachelor’s Degree*	−0.04	0.09 *	0.03	0.17 ***	−0.01	0.14 *
	(−0.10, 0.03)	(0.01, 0.16)	(−0.06, 0.12)	(0.08, 0.26)	(−0.15, 0.13)	(0.02, 0.25)

^a^ All models adjusted for sex, age, income, occupation, subjective social ranking, and individual coronavirus exposure. ^b^ Models additionally adjusted for the perceived risk of outbreak and interventional context. ^c^ Models additionally adjusted for context cues of danger and interventional context. ^d^ Models additionally adjusted for context cues of danger and perceived risk of an outbreak. 95% confidence intervals in brackets. * *p* < 0.05, ** *p* < 0.01, *** *p* < 0.001.

**Table 4:** Educational Disparities in COVID-19 Preventive Behaviors Stratified by Context Cues of Danger, The Subsample Excluding Wuhan Residents, The Chinese Survey of COVID-19 Impacts 2020 (*N* = 2289).

Context Cue of Danger	Mask Wearing ^a^		Handwashing ^a^		Limited Public Outing ^a^	
	Present (*N* = 1846)	Absent (*N* = 443)	Present (*N* = 1846)	Absent (*N* = 443)	Present (*N* = 1846)	Absent (*N* = 443)
Intercept	3.51 ***	3.65 ***	3.37 ***	3.25 ***	3.32 ***	3.22 ***
	(3.36, 3.66)	(3.38, 3.93)	(3.22, 3.53)	(2.92, 3.59)	(3.13, 3.51)	(2.79, 3.65)
Education ^b^						
*≤High School*						
*Associate Degree*	−0.01	0.23 **	0.05	0.07	−0.02	0.28 *
	(−0.09, 0.08)	(0.09, 0.37)	(−0.04, 0.14)	(−0.10, 0.24)	(−0.13, 0.09)	(0.06, 0.51)
*≥Bachelor’s Degree*	0.04	0.31 ***	0.13 **	0.22 *	0.09	0.28 *
	(−0.05, 0.12)	(0.17, 0.44)	(0.04, 0.22)	(0.05, 0.39)	(−0.02, 0.19)	(0.06, 0.49)
**Perceived Risk of Local Outbreak**	Zero/Low (*N* = 2051)	Medium/High (*N* = 238)	Zero/Low (*N* = 2051)	Medium/High (*N* = 238)	Zero/Low (*N* = 2051)	Medium/High (*N* = 238)
Intercept	3.52 ***	3.48 ***	3.36 ***	3.12 ***	3.24 ***	3.55 ***
	(3.37, 3.66)	(3.13, 3.84)	(3.22, 3.51)	(2.63, 3.60)	(3.05, 3.42)	(2.95, 4.15)
Education ^c^						
*≤High School*						
*Associate Degree*	0.01	0.33 **	0.02	0.40 **	0	0.50 **
	(−0.07, 0.09)	(0.12, 0.54)	(−0.06, 0.10)	(0.12, 0.68)	(−0.10, 0.10)	(0.16, 0.85)
*≥Bachelor’s Degree*	0.05	0.35 ***	0.11 **	0.45 **	0.09	0.46 **
	(−0.02, 0.13)	(0.14, 0.55)	(0.03, 0.19)	(0.18, 0.72)	(−0.01, 0.19)	(0.12, 0.79)

^a^ All models adjusted for sex, age, urbanicity, income, occupation, subjective social ranking, and individual coronavirus exposure. ^b^ Models additionally adjusted for the perceived risk of an outbreak. ^c^ Models additionally adjusted for context cue of danger. 95% confidence intervals in brackets. * *p* < 0.05, ** *p* < 0.01, *** *p* < 0.001.

## Supplementary Materials

The following are available online at https://www.mdpi.com/1660-4601/18/7/3383/s1, Table S1. Models based on multiply imputed data, The Chinese Survey of COVID-19 Impacts 2020 (*N* = 4638). Figure S1. Confirmed COVID-19 Cases by Province by the Time of Survey.

## Appendix A

**Table A1:** Survey Questions for Relevant Variables.

Variable Name	Survey Question	Coding
**Outcomes**		
Mask wearing in public	In order to prevent the coronavirus infection, are you currently practicing the following things: wear a mask when you go out	5-point scale, 1 = not at all, 5 = all the time
Handwashing after a public outing	In order to prevent the coronavirus infection, are you currently practicing the following things: wash your hand when you return from outside	5-point scale, 1 = not at all, 5 = all the time
Limited public outing	In order to prevent the coronavirus infection, are you currently practicing the following things: avoid unnecessary outing	5-point scale, 1 = not at all, 5 = all the time
**Independent Variable**		
Education	What is your highest degree in education?	1 = high school or lower, 2 = associate degree, 3 = bachelor’s degree or above
**Moderator**		
Contextual cues of danger	During the coronavirus outbreak, whether any of your family members were confirmed to have COVID-19?	0 = no, 1 = yes
	During the coronavirus outbreak, whether any of your family members were quarantined/hospitalized due to suspected coronavirus positivity or close contact with someone with confirmed positivity?	0 = no, 1 = yes
	During the coronavirus outbreak, whether there are confirmed COVID-19 cases in the neighborhood/village that you are living in?	0 = no, 1 = yes
Perceived risk of an outbreak	In your opinion, what is the coronavirus outbreak risk in the place you live? Is it high, medium, low, or none?	0 = none or low, 1 = medium or high
Interventional context	Which city are you currently living in?	0 =all other places, 1 = Wuhan
**Covariates**		
Sex	What is your sex?	0 = female, 1 = male
Age	Which year were you born?	in years
Urbanicity	Are you currently living in the urban area or countryside?	0 = rural, 1 = urban
Income	In 2019, what is the average income for each person within your household (that is, the total income divided by the number of family members)?	in logged ten-thousand Ұ
Occupation	What is your occupation?	1 = executives or professionals, 2 = unskilled or low-skilled labor, 3 = currently not in the labor force, 4 = other
Self-rated social ranking	In comparison to other people in the area you live, do you consider your family as the upper class, upper-middle class, middle class, lower-middle class, or lower class.	5-point scale, 1 = lower class, 5 = upper class
Individual coronavirus exposure	During the coronavirus outbreak, were you confirmed to have COVID-19?	0 = no, 1 = yes
	During the coronavirus outbreak, were you quarantined/hospitalized due to suspected coronavirus positivity or close contact with someone with confirmed positivity?	0 = no, 1 = yes

## References

[1] Lieberman-Cribbin W., Tuminello S., Flores R.M., Taioli E. (2020). Disparities in COVID-19 Testing and Positivity in New York City. Am. J. Prev. Med..

[2] Karaye I.M., Horney J.A. (2020). The Impact of Social Vulnerability on COVID-19 in the U.S.: An Analysis of Spatially Varying Relationships. Am. J. Prev. Med..

[3] Webb Hooper M., Nápoles A.M., Pérez-Stable E.J. (2020). COVID-19 and Racial/Ethnic Disparities. JAMA.

[4] Lewnard J.A., Lo N.C. (2020). Scientific and ethical basis for social-distancing interventions against COVID-19. Lancet Infect. Dis..

[5] Sunjaya A.P., Jenkins C. (2020). Rationale for universal face masks in public against COVID-19. Respirology.

[6] Clouston S.A.P., Richards M., Cadar D., Hofer S.M. (2015). Educational Inequalities in Health Behaviors at Midlife: Is There a Role for Early-life Cognition?. J. Health Soc. Behav..

[7] Brunello G., Fort M., Schneeweis N., Winter-Ebmer R. (2016). The Causal Effect of Education on Health: What is the Role of Health Behaviors?. Health Econ..

[8] Hecht E.M., Layton M.R., Abrams G.A., Rabil A.M., Landy D.C. (2020). Healthy Behavior Adherence: The National Health and Nutrition Examination Survey, 2005–2016. Am. J. Prev. Med..

[9] Shankar A., McMunn A., Steptoe A. (2010). Health-Related Behaviors in Older Adults: Relationships with Socioeconomic Status. Am. J. Prev. Med..

[10] Link B.G., Phelan J. (1995). Social Conditions as Fundamental Causes of Disease. J. Health Soc. Behav..

[11] Ross C.E., Mirowsky J. (2006). Sex differences in the effect of education on depression: Resource multiplication or resource substitution?. Soc. Sci. Med..

[12] Ross C.E., Mirowsky J. (2011). The interaction of personal and parental education on health. Soc. Sci. Med..

[13] Sen A. (1997). Editorial: Human capital and human capability. World Dev..

[14] Mirowsky J., Ross C.E. (2015). Education, Health, and the Default American Lifestyle. J. Health Soc. Behav..

[15] Lawrence E.M. (2017). Why Do College Graduates Behave More Healthfully Than Those Who Are Less Educated?. J. Health Soc. Behav..

[16] Baker D.P., Smith W.C., Muñoz I.G., Jeon H., Fu T., Leon J., Salinas D., Horvatek R. (2017). The Population Education Transition Curve: Education Gradients across Population Exposure to New Health Risks. Demography.

[17] Baker D.P., Leon J., Collins J.M. (2011). Facts, Attitudes, and Health Reasoning About HIV and AIDS: Explaining the Education Effect on Condom Use Among Adults in Sub-Saharan Africa. Aids Behav..

[18] Zajacova A., Lawrence E.M. (2018). The Relationship between Education and Health: Reducing Disparities through a Contextual Approach. Annu. Rev. Public Health.

[19] Cutler D.M., Lleras-Muney A. (2010). Understanding differences in health behaviors by education. J. Health Econ..

[20] Pampel F.C., Krueger P.M., Denney J.T. (2010). Socioeconomic Disparities in Health Behaviors. Annu. Rev. Sociol..

[21] Phelan J.C., Link B.G., Tehranifar P. (2010). Social Conditions as Fundamental Causes of Health Inequalities: Theory, Evidence, and Policy Implications. J. Health Soc. Behav..

[22] Link B.G. (2008). Epidemiological Sociology and the Social Shaping of Population Health. J. Health Soc. Behav..

[23] Pampel F.C. (2005). Diffusion, cohort change, and social patterns of smoking. Soc. Sci. Res..

[24] de Walque D. (2010). Education, Information, and Smoking Decisions: Evidence from Smoking Histories in the United States, 1940–2000. J. Hum. Resour..

[25] Miech R. (2008). The Formation of a Socioeconomic Health Disparity: The Case of Cocaine Use during the 1980s and 1990s. J. Health Soc. Behav..

[26] Miech R.A., Chilcoat H., Harder V.S. (2005). The increase in the association of education and cocaine use over the 1980s and 1990s: Evidence for a ’historical period’ effect. Drug Alcohol Depend..

[27] Margolis R. (2013). Educational Differences in Healthy Behavior Changes and Adherence among Middle-aged Americans. J. Health Soc. Behav..

[28] Duberstein P.R., Chen M., Chapman B.P., Hoerger M., Saeed F., Guancial E., Mack J.W. (2018). Fatalism and educational disparities in beliefs about the curability of advanced cancer. Patient Educ. Couns..

[29] Lawlor D.A., Frankel S., Shaw M., Ebrahim S., Smith G.D. (2003). Smoking and ill health: Does lay epidemiology explain the failure of smoking cessation programs among deprived populations?. Am. J. Public Health.

[30] Ajzen I. (1991). The Theory of Planned Behavior. Organ. Behav. Hum. Decis. Process..

[31] Mackenbach J.P., Kulhánová I., Bopp M., Deboosere P., Eikemo T.A., Hoffmann R., Kulik M.C., Leinsalu M., Martikainen P., Menvielle G. (2015). Variations in the relation between education and cause-specific mortality in 19 European populations: A test of the “fundamental causes” theory of social inequalities in health. Soc. Sci. Med..

[32] Masters R.K., Link B.G., Phelan J.C. (2015). Trends in education gradients of ‘preventable’ mortality: A test of fundamental cause theory. Soc. Sci. Med..

[33] Rydland H.T., Solheim E.F., Eikemo T.A. (2020). Educational inequalities in high- vs. low-preventable health conditions: Exploring the fundamental cause theory. Soc. Sci. Med..

[34] Hoynes H., Miller D., Simon D. (2015). Income, the Earned Income Tax Credit, and Infant Health. Am. Econ. J. Econ. Policy.

[35] Wendel-Vos G.C.W., Dutman A.E., Verschuren W.M.M., Ronckers E.T., Ament A., van Assema P., van Ree J., Ruland E.C., Schuit A.J. (2009). Lifestyle Factors of a Five-Year Community-Intervention Program: The Hartslag Limburg Intervention. Am. J. Prev. Med..

[36] Pan A., Liu L., Wang C., Guo H., Hao X., Wang Q., Huang J., He N., Yu H., Lin X. (2020). Association of Public Health Interventions With the Epidemiology of the COVID-19 Outbreak in Wuhan, China. JAMA.

[37] World Health Organization (2020). Report of the WHO-China Joint Mission on Coronavirus Disease 2019 (COVID-19).

[38] Ministry of Industry and Information Technology of China. Briefing on the Major Development Indicators in the Communications Industry: Janurary–June 2020. http://www.miit.gov.cn/n1146312/n1146904/n1648372/c8021317/content.html.

[39] Cai J., Coyte P.C., Zhao H. (2017). Determinants of and socio-economic disparities in self-rated health in China. Int. J. Equity Health.

[40] Liu J., Zhang Y. (2019). Health status and health disparity in China: A demographic and socioeconomic perspective. China Popul. Dev. Stud..

[41] Zhou X. (2014). Increasing Returns to Education, Changing Labor Force Structure, and the Rise of Earnings Inequality in Urban China, 1996–2010. Soc. Forces.

[42] Lüdecke D., von dem Knesebeck O. (2020). Protective Behavior in Course of the COVID-19 Outbreak—Survey Results from Germany. Front. Public Health.

[43] Al-Hanawi M.K., Angawi K., Alshareef N., Qattan A.M.N., Helmy H.Z., Abudawood Y., Alqurashi M., Kattan W.M., Kadasah N.A., Chirwa G.C. (2020). Knowledge, Attitude and Practice Toward COVID-19 among the Public in the Kingdom of Saudi Arabia: A Cross-Sectional Study. Front. Public Health.

[44] Groves R.M., Peytcheva E. (2008). The Impact of Nonresponse Rates on Nonresponse Bias: A Meta-Analysis. Public Opin. Q..

