# Supplementary material for: Educational Disparities in COVID-19 Prevention in China: The Role of Contextual Danger, Perceived Risk, and Interventional Context
Source: Int J Environ Res Public Health. 2021 Mar 24;18(7):3383. doi: 10.3390/ijerph18073383 (PMC8036684; doi:10.3390/ijerph18073383)

Table S1. Educational Disparities in COVID-19 Preventive Behaviors Stratified by Context Cue of Danger, Perceived Risk of Local Outbreak, and Interventional Context, The Chinese Survey of COVID-19 Impacts 2020 (N = 4638) [a]

	Mask Wearing [b]		Handwashing b		Limited Public Outing [b]	
**Context Cue of Danger**	No (N = 3098)	Yes (N = 1540)	No (N = 3098)	Yes (N = 1540)	No (N = 3098)	Yes (N = 1540)
Intercept	3.55 *** (3.43,3.67)	3.60 *** (3.46,3.73)	3.36 *** (3.24,3.49)	3.11 *** (2.95,3.28)	3.30 *** (3.15,3.46)	3.02 *** (2.77,3.27)
Education [c]						
≤High School						
Associate Degree	0.04 (−0.02,0.11)	0.08 * (0.02,0.15)	0.05 (−0.01,0.12)	0.08 * (0.00,0.16)	0 (−0.08,0.08)	0.18 ** (0.06,0.29)
≥Bachelor's Degree	0.03 (−0.03,0.09)	0.13 *** (0.07,0.19)	0.07 * (0.01,0.14)	0.14 *** (0.06,0.22)	0.04 (−0.04,0.13)	0.13 * (0.02,0.24)
**Perceived Risk of Local Outbreak**	Zero/Low (N = 4024)	Medium/High (N = 614)	Zero/Low (N = 4024)	Medium/High (N = 614)	Zero/Low (N = 4024)	Medium/High (N = 614)
Intercept	3.55 *** (3.44,3.66)	3.43 *** (3.20,3.65)	3.31 *** (3.19,3.42)	3.14 *** (2.84,3.44)	3.18 *** (3.03,3.33)	3.16 *** (2.74,3.59)
Education [d]						
≤ High School						
Associate Degree	0.04 (−0.02,0.09)	0.21 *** (0.10,0.32)	0.05 (−0.00,0.11)	0.12 (−0.03,0.28)	0.02 (−0.05,0.10)	0.32 ** (0.12,0.53)
≥ Bachelor's Degree	0.04 (−0.01,0.09)	0.21 *** (0.10,0.31)	0.09 *** (0.04,0.15)	0.16 * (0.02,0.30)	0.03 (−0.04,0.11)	0.36 *** (0.16,0.55)
**Interventional Context**	Wuhan (N = 1379)	Other Areas (N = 3259)	Wuhan (N = 1379)	Other Areas (N = 3259)	Wuhan (N = 1379)	Other Areas (N = 3259)
Intercept	3.69 *** (3.57,3.82)	3.53 *** (3.41,3.65)	3.21 *** (3.05,3.38)	3.34 *** (3.21,3.46)	3.20 *** (2.95,3.44)	3.25 *** (3.09,3.41)
Education e						
≤High School						
Associate Degree	0.03 (−0.02,0.09)	0.07 * (0.00,0.13)	0.08 (0.00,0.16)	0.05 (−0.01,0.12)	0.08 (−0.04,0.19)	0.05 (−0.03,0.14)
≥Bachelor's Degree	0.02 (−0.04,0.07)	0.08 * (0.02,0.14)	0.07 (−0.01,0.15)	0.11*** (0.05,0.18)	0.04 (−0.07,0.16)	0.08 * (0.00,0.17)

[a] Fifteen cases with missing data on outcomes were excluded.[b] All models adjusted for sex, age, urbanicity, income, occupation, subjective social ranking, and individual coronavirus exposure.[c] Models additionally adjusted for perceived risk of outbreak and interventional context.[d] Models additionally adjusted for context cue of danger and interventional context. e Models additionally adjusted for context cue of danger and perceived risk of outbreak. 95% confidence intervals in brackets. * $p < 0.05$, ** $p < 0.01$, *** $p < 0.001$.

Figure S1. Confirmed COVID-19 Cases By Province By the Time of Survey

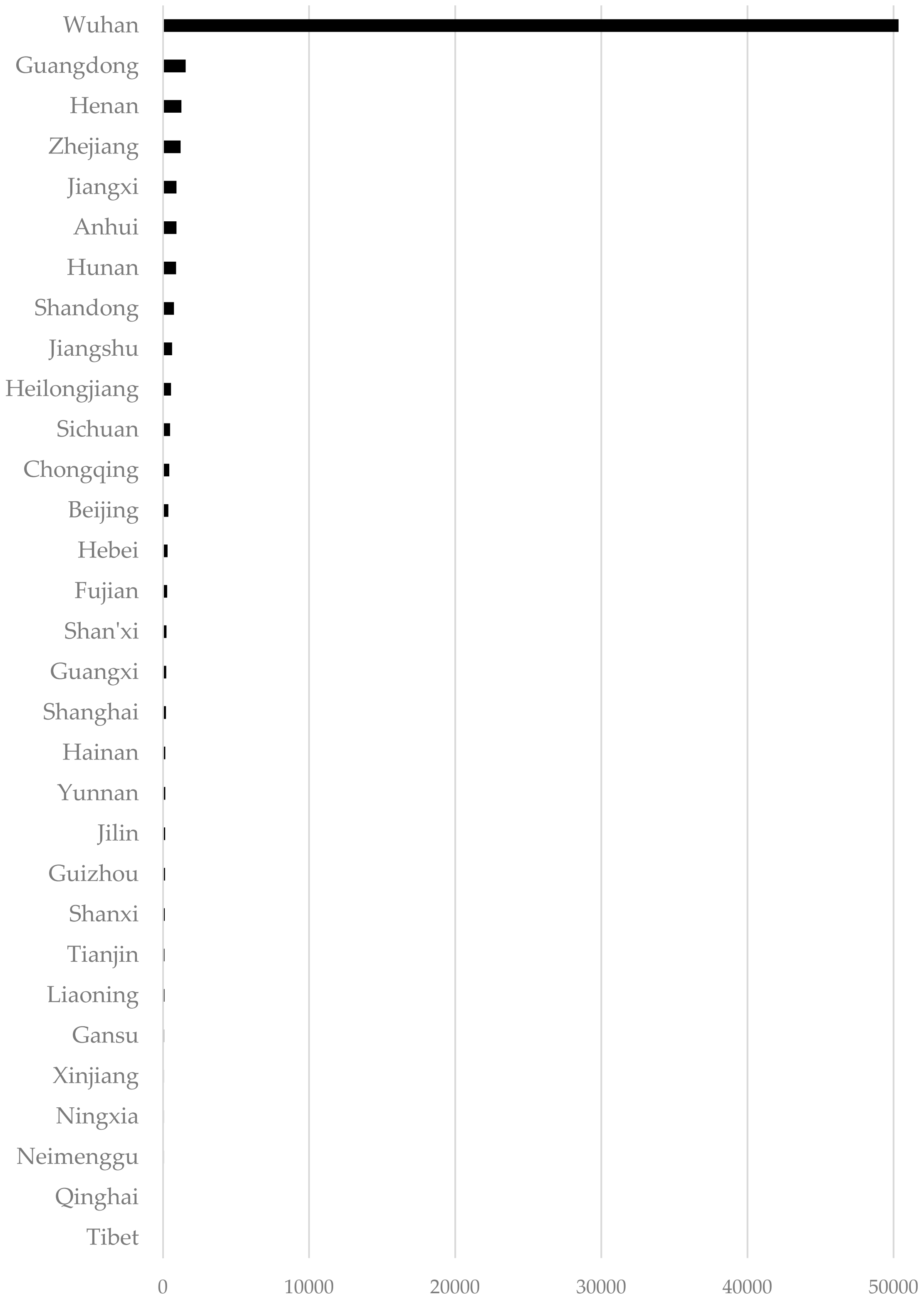

Supplement: Supplementary file 1 [file ijerph-18-03383-s001.pdf]
